# The Antioxidant, Antibacterial and Anti-Biofilm Properties of Rapeseed Creamed Honey Enriched with Selected Plant Superfoods

**DOI:** 10.3390/antibiotics12020235

**Published:** 2023-01-22

**Authors:** Michał Miłek, Ewa Ciszkowicz, Ewelina Sidor, Joanna Hęclik, Katarzyna Lecka-Szlachta, Małgorzata Dżugan

**Affiliations:** 1 Department of Chemistry and Food Toxicology, Institute of Food Technology and Nutrition, University of Rzeszów, Ćwiklinskiej 1a, 35-601 Rzeszów, Poland; 2 Department of Biotechnology and Bioinformatics, Faculty of Chemistry, Rzeszow University of Technology, Powstańców Warszawy 6, 35-959 Rzeszów, Poland; 3 Doctoral School, University of Rzeszów, Rejtana 16c, 35-959 Rzeszow, Poland

**Keywords:** honey, superfruits, superherbs, antioxidant, antibacterial, anti-biofilm

## Abstract

The aim of the study is to evaluate the effect of the addition of selected fruits and herbs belonging to the “superfoods” category for the bioactivity of a rapeseed honey matrix. Flavored creamed honeys with nine types of various additives (2 and 4% of content) were prepared and analyzed for the content of total phenols, flavonoids, antioxidant (FRAP, DPPH and ABTS) and antibacterial activity against four strains of bacteria. Additionally, the impact of three months of storage on the antioxidant properties of the products obtained was examined. The significant dose-dependent increase in the content of bioactive ingredients and antioxidant capacity in spiced honeys, as compared to control honey, was observed. The highest enrichment was obtained for the addition of powdered sea buckthorn leaves and black raspberry fruits. Honey with the addition of sea buckthorn leaves inhibited the growth of *P. aeruginosa*, *S. aureus* and *K. pneumonia,* whereas honeys with black raspberry and blackcurrant fruits showed activity only on the latter two strains. Furthermore, what is more interesting, honey supplemented with sea buckthorn leaf and black raspberry fruits inhibited *S. aureus* biofilm formation at the sub-minimum inhibitory concentrations (sub-MICs), showing a dose-dependent anti-biofilm effect.

## 1. Introduction

Apart from traditional honey, royal jelly, propolis or pollen, the market for bee products is still open to innovations. Herbal honeys and creamed honeys with various additives such as fruit, herbs, and spices are becoming more and more popular with consumers. Consequently, it is causing an increase in the supply of such products and the search for new flavor variants. A few years ago, honey with the addition of other bee products, such as propolis, pollen and bee bread appeared on the Polish market and the research into such products began [1,2,3,4]. Later, numerous variants of mixtures of plants and honeys were introduced: with spices [5,6,7], herbs and fruit [6,8,9,10,11,12] and even with essential oils [13]. Such products are also named differently as “enriched” [6,8,9,10,11,12], ”flavored” [14], “spiced” [15], “infused” [16] or “with added value” [17] honeys. They all share a combination of the valuable properties of honey with the beneficial properties of fruit, fruit juices or medicinal plants mixed in various proportions.

The introduction of a plant additive to honey, in addition to shaping more favorable sensory properties, i.e., color, taste or smell, significantly increases its bioactivity. Bee honey itself is known for its antioxidant and antimicrobial properties. Its antiradical and reducing properties are mainly determined by the presence of phenolic compounds. So far, several dozen compounds from different classes of polyphenols have been detected in various types of honey, mainly phenolic acids and flavonoids [18,19]. The antibacterial properties of honey, in turn, are the result of several mechanisms: high sugar content (osmotic pressure), low pH value (3.2–4.5), hydrogen peroxide generated by glucose oxidase (catalyzing reaction of glucose oxidation), the presence of antibacterial peptides (e.g., defensin-1) and polyphenolic compounds [19,20]. What is more, there are several studies that have proven that the introduction of plant additives to honey causes a significant increase in antioxidant and antimicrobial potential against selected bacteria, yeasts and viruses [8,10,11,12]. This enhancement is explained as being a result of the synergy between the active ingredients of honey and medicinal plants [8,12]. The search for such combinations that, while attractive to consumers, will show an exceptionally strong pro-health effect and may serve as functional food included in the diet, is still ongoing.

Our attempts to prepare creamed honeys with additives, the so-called “superfruits” or “superherbs”, aim to discover new effective combinations that are either not yet available on the market or not very popular. The fruits used, including black raspberry, black currant, rosehip, barberry and sea buckthorn, are extremely rich in vitamins and polyphenolic compounds, mainly anthocyanins. They have frequently demonstrated strong pharmacological action and beneficial nutritional value [21,22,23,24,25,26]. In addition, elderberry flowers were used, known for their antioxidant, anti-inflammatory and antimicrobial properties [27,28], as well as sea buckthorn leaves, which are an underestimated by-product of valuable fruit production [29,30].

The aim of the following study was to prepare creamed honey enriched with nine plant additives and to evaluate the influence of such a combination on the antioxidant, antibacterial and anti-biofilm properties of the honey matrix. Additionally, the shelf stability of these products was tested after three months of storage.

## 2. Results and Discussion

The prepared creamed honeys were evaluated for the content of total phenolic and flavonoid fraction (Table 1). In the control rapeseed honey, 35.56 mg GAE/100 g was determined, which was earlier found to be typical for this honey variety [31,32]. Rapeseed honey, which has been selected as the base, is known to have the weakest biological activity among Polish honey varieties [32]. Moreover, this honey shows the fastest rate of crystallization (a few days after harvesting) among all the varieties of monofloral honeys; therefore it is the best for creaming, especially when mixed with solid additives [33].

As a result of enrichment with plant material, a significant increase in the content of phenolic compounds was obtained, ranging from 15% (2% addition of apple powder) to a greater than seventeen-fold increase (4% addition of sea buckthorn leaves). A similar degree of enrichment was observed for the total flavonoid content, with the highest concentration of these compounds found in honey with the addition of sea buckthorn fruit. While fruits of sea buckthorn (*Hippophae rhamnoides*) are well known for their high content of polyphenols, including flavonoids, the leaves of this plant are also abundant in such components [25,26,30,34]. The observed multiplied content of polyphenols was reported in our previous studies where enrichment in plant-origin polyphenolic compounds profiled by chromatographic methods for honeys enriched with mulberry [10], chokeberry [11], as well as blackberry and raspberry [12] was recorded. 

The antioxidant activity measured by the DPPH, ABTS and FRAP methods for the control rapeseed honey was at a level typical for this honey variety [10,32]. Plant additives introduced into the raw honey caused a substantial increase in its reducing power and antiradical capacity (Table 1). The strongest increase was noted in the case of sea buckthorn leaves, together with wild rose, black raspberry, and blackcurrant fruit additives. These fruits contain numerous antioxidants, beside flavonoids and phenolic acids, as well as anthocyanins and vitamin C [25,26,35,36]). Moreover, a high correlation between total phenolic content and antioxidant capacity was observed (r = 0.985, 0.954, 0.872 for TPC vs. FRAP, DPPH and ABTS, respectively). However, there was no significant correlation between flavonoid content and antioxidant activity regardless of the applied assay (r below 0.4). Similarly, a weaker correlation of flavonoids content with antioxidants capacity was observed in honey enriched with *Rubus* spp. [12].

The amount of the additive introduced into the honey was significant: the higher the share of the plant material, the higher the enrichment in bioactive components that was observed. This correlation is in line with our previous studies on similar products [11,12]. Among the additives used, only *Rosa* spp. fruits were previously tested as an additive to acacia honey [8]. The introduction of 5 and 10% rose hips resulted in a more than two-fold increase in the antiradical activity in the DPPH assay; however, with a lower number of fruits the OH-antiradical power increased by four and more than six times, respectively. The increase in the antioxidant properties of honey with the addition of fruit is explained by synergistic interactions between antioxidants of honey and those present in plant material: mainly polyphenols and vitamin C [8,12]. Recently, 20 flavored honeys commercially available in Polish retail shops have been studied [14], including honey enriched with sea buckthorn berries (2%). However, these commercial honeys were evaluated only in terms of physicochemical parameters and antibacterial activity rather than antioxidant capacity, which makes a comparison with the present study impossible.

**Figure 1:**
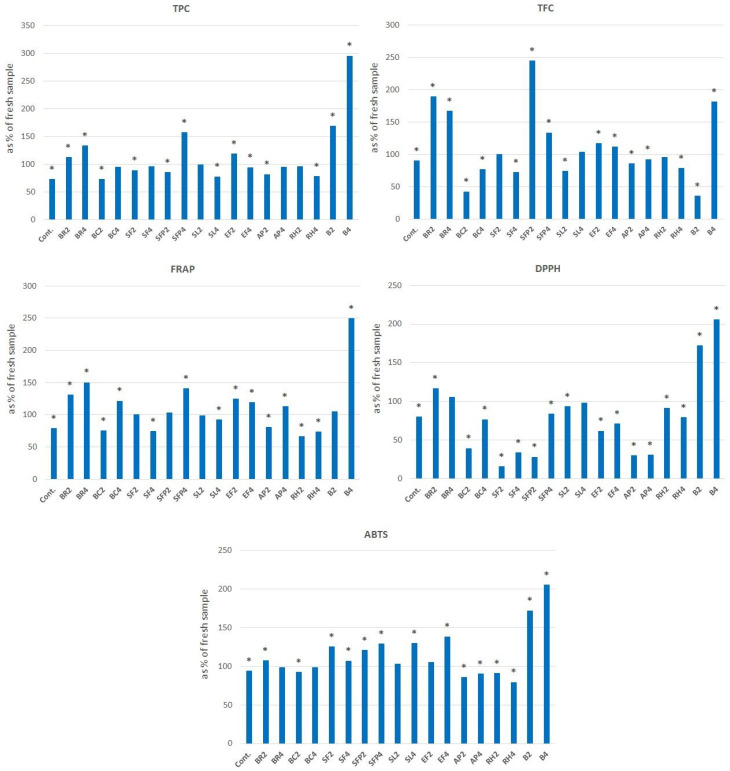
Percentage changes in the tested parameter related to antioxidant activity determined after three months of storage of flavored honeys in relation to the initial value (fresh sample). Cont.—control honey, BR—black raspberry, BC—blackcurrant, SF—sea buckthorn fruit, SFP—sea buckthorn fruit powder, SFL—sea buckthorn leaf, EF—elderberry flower, AP—apple powder, RH—rose hips, B—barberry, *—a statistically significant difference compared to the fresh sample (*p* > 0.05).

In order to determine the storage stability of the obtained products, the antioxidant activity tests were repeated after three months of storage at room temperature (Figure 1). The stability of the antioxidant activity and polyphenol content differed significantly, depending on the additive used. In some cases, significant decreases (up to 71 and 34% in DPPH and FRAP assays, respectively) in the antioxidant properties of flavored honeys were observed. In others, a strong increase (up to 150%, measured by FRAP assay) in tested parameters was noted. The greatest increase was observed for honey with a higher share of barberry fruits where the tested parameters reached even a two- to three-fold increase in time. Different behavior can be explained by the different migration of bioactive components into the honey matrix, depending probably on the morphology of the plant additives used, i.e., fruit, leaf or flower particles. Similarly, diverse changes in TPC and FRAP during storage were previously observed for honeys creamed with fruits and leaves of *Rubus* spp. [12].

The antibacterial activity of honey samples with a higher share of the additive (4%) was evaluated by determining their minimum inhibitory concentrations (MIC) (Table 2). Compared to the commercially available antibiotics, all prepared honeys demonstrated poor activities against all tested strains. No antibacterial activity was observed against *E. coli*. Honey with the addition of sea buckthorn leaf (4%) revealed the broadest spectrum activity against the other bacterial strains with MIC = 250 mg/mL and MIC = 125 mg/mL against *P. aeruginosa* and *S. aureus/K. pneumoniae*, respectively. The highest identified phenolic content (616.82 mg/100 g), as well as antioxidant activity confirmed by the three performed methods (FRAP, DPPH and ABTS), explains the effectiveness of honey with a 4% addition of sea buckthorn leaf against tested bacteria. The results obtained confirm numerous reports on the antibacterial activity of sea buckthorn [37,38]; however, they contradict the research of the authors who observed strong antibacterial activity against *E. coli* [39]. The addition of black raspberry (4%) and blackcurrant (4%) to the honey resulted in the acquisition of antibacterial activity of the respective honeys against *S. aureus* and *K. pneumoniae*. The introduction of these fruits into honey resulted in the appearance of anthocyanins in the product, in which blackcurrant and black raspberry are particularly abundant. These compounds are known to have antibacterial activity against a variety of bacterial strains, including *Staphylococcus* sp. and *Klebsiella pneumoniae* [40]. In turn, sea buckthorn leaves containing myricetin, luteolin, vitexin, kaempferol, numerous quercetin derivatives, and phenolic acids, showed activity only against some bacteria, e.g., *B. cereus, P. aeruginosa* and *S. aureus* [25].

Rapeseed honey selected as the base is known for its minimal effect on microorganisms (with MIC > 25% *v/v*) [41]. Therefore, the intention of introducing plant additives to honey of this particular variety is to significantly increase its beneficial properties, including antioxidant and antimicrobial activity. This aim has been achieved to some extent.

The bactericidal or bacteriostatic type of action was evaluated by attempting to identify MBC. The type of action was determined as bactericidal for black raspberry (4%) and blackcurrant (4%) with MBC = MIC = 250 mg/mL (i.e., 25% *w/v*) against *K. pneumoniae*. Regarding *S. aureus,* three honey samples (black raspberry (4%), blackcurrant (4%) and sea buckthorn leaf (4%)) with identified antibacterial activity demonstrated a bacteriostatic mode of action. The significant effects that the type of plant additive combined with honey has on increasing its antibacterial activity were demonstrated by Żebracka et al. (2022) [14], who studied the bactericidal properties of commercial flavored honeys available on the Polish market. It was noted that nectar honey with flavorings showed statistically significant differences in the case of *E. coli* for honeys enriched with marshmallow root and elderberry; in the case of *S. enteritidis* for honey with the addition of orange paste and cloves; and in the case of *S. aureus* for honey mixed with concentrated lemon juice and honey with cranberry (*p* < 0.05). Flavored honey had no effect on *Pseudomonas aeruginosa*, irrespective of the added flavorings.

**Figure 2:**
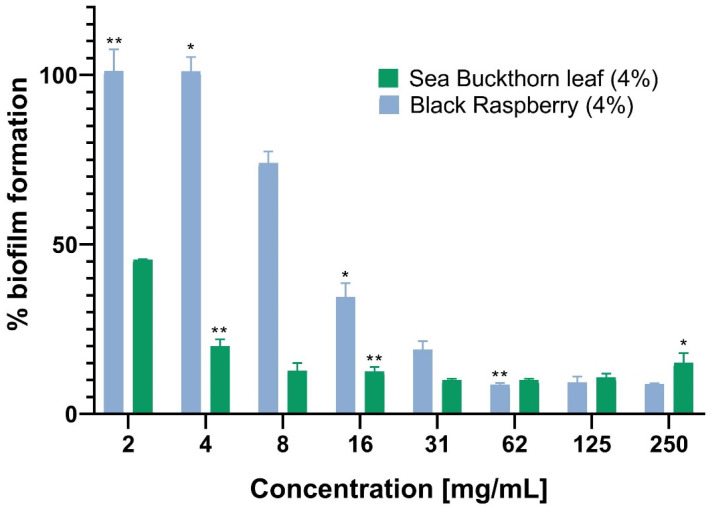
Antibiofilm activity of honey samples in two-fold decreasing concentrations against certified *S. aureus*. The results were determined in relation to a non-treated control (100% of biofilm formation). The error bars and asterisks represent the standard errors and statistical significance with the *p*-values: ** *p* < 0.01 and * *p* < 0.05.

The effects of prepared honeys on the inhibition of biofilm formation were also investigated (Figure 2). The biofilm of the certified *S. aureus* strain was grown in the presence of a decreasing concentration of honeys. The results revealed that only black raspberry (4%) and sea buckthorn (4%) were able to inhibit the formation of *S. aureus* biofilm in a concentration-related manner. The amount of biofilm formed by *S. aureus* was affected differently after exposure to the two aforementioned honeys (Figure 2).

Honey with the addition of sea buckthorn leaf (4%) was the most active in inhibiting biofilm formation. At the lowest tested concentration of 2 mg/mL, it inhibited as much as 65% of the biofilm, as compared to the *S. aureus* control with no antibacterial agent.

Our results confirm the antibacterial properties of sea buckthorn leaves that have already been reported against bacterial strains that cause serious food poisoning (*Bacillus subtilis, Listeria monocytogenes, Salmonella enterica*) [37] and infections *(S. aureus, Pseudomonas aeruginosa*) [42,43]. The addition of black raspberry (4%) to the honey sample also resulted in enhanced anti-biofilm activity, leading to approximately 70% inhibition of biofilm formation at a concentration of 16 mg/mL. Both sea buckthorn and black raspberry are extremely rich in polyphenolic compounds, including anthocyanins [44], which may result in a high antibacterial activity of honeys with the addition of these two extracts.

The effect of similar products (rapeseed honey with raspberry and blackberry fruit and leaves) on the *S. aureus* ATCC 25923 biofilm has previously been studied. The inhibition of the biofilm formation was shown to be more effective than pre-established biofilm elimination, especially for honeys with the addition of *Rubus* spp. leaves [12]. It has also been implied that sub-MICs of certain antimicrobial agents can suppress the formation of biofilms by disrupting the adhering capacity [45]; however, their higher concentrations may result in the induction of resistance [46]. Hence, concentrations of antimicrobial agents may influence the bacterial virulence parameters such as adherence, motility, biofilm formation and sensitivity to oxidative stress [47,48,49]. The mechanism of the anti-biofilm action of tested enriched honeys needs to be further investigated.

## 3. Materials and Methods

### 3.1. Honey and Plant Additives

Fresh rapeseed honey was obtained from the local apiary located in the Subcarpathia region (50.31° N, 21.28° E) during the beekeeping season. Freeze-dried fruits: black raspberry (*Rubus occidentalis*), blackcurrant (*Ribes nigrum*), wild rose hips (*Rosa canina*), barberry (*Berberis vulgaris*), as well as dried elderberry flower (*Sambucus nigra*) were purchased at a local health food store. Dried fruits, micronized dried fruit powder and dried leaves of sea buckthorn (*Hippophae rhamnoides*), as well as micronized dried apple powder (*Malus domestica),* were obtained from BiGrim Company (Wojciechów, Poland).

### 3.2. Preparation of Enriched Honey Samples

At first, the honey was completely decrystallized by heating it up to 42 °C for 48 h using a laboratory incubator (SLN 53 STD, Pol Eko, Wodzisław Śląski, Poland). The liquefied honey was inoculated with crystalized honey (99:1 g) and mechanically mixed for 60 s four times a day. Pulverized plant additives were then introduced to the honey (2 and 4%, *w/w*) and mixed. The samples were stored at 4 °C for three days and mixed twice a day. Subsequently, honeys were stored for 30 days at room temperature (20 ± 2 °C) in the dark until the crystallization process was complete and were later subjected to analysis. The analysis was repeated after 90 days of storage.

### 3.3. Total Phenolic Content (TPC) and Total Flavonoid Content (TFC)

The total phenolic content and the total flavonoid content were measured for 20% solutions of honey in distilled water, as described by Miłek et al. (2021) [11]. Results were expressed as mg of gallic acid equivalents (GAE) and quercetin equivalents (QE) per 100 g of sample, respectively.

### 3.4. Antioxidant Capacity

Antioxidant capacity was measured in 20% solutions of honeys in distilled water in three assays: a ferric-reducing antioxidant powder (FRAP), a DPPH radical scavenging test, and an ABTS radical cation scavenging test. The assays were performed as described by Miłek et al. (2021) [11]. The results were expressed as Trolox equivalents (TE) per 100 g of sample using appropriate calibration curves.

### 3.5. Antibacterial Effect

The antibacterial properties of honeys with a 4% addition of selected fruits and herbs were determined. Minimum inhibitory concentration (MIC) and minimum bactericidal concentration (MBC) methods against Gram-negative (*Escherichia coli* ATCC 10536, *Pseudomonas aeruginosa* ATCC 15442, *Klebsiella pneumoniae* ATCC 13883) and Gram-positive bacteria (*Staphylococcus aureus* ATCC 6538) were used [50]. Inhibition of *S. aureus* biofilm formation was performed according to Bocian et al. (2020) [51]. The prepared honeys and antibiotics (chloramphenicol and gentamicin) were tested in the concentration range, respectively, from 1.95 to 250 mg/mL and from 0.2 × 10^−3^ to 0.5 mg/mL. The MIC was defined as the lowest concentration of the antibacterial agent, which completely inhibited the visible growth of the microorganism, and the MBC was determined as the lowest concentration at which there was no bacterial growth on agar plates after additional 24 h incubation at 37 °C [52]. No inhibition of bacterial growth indicates the bacteriostatic type of action of the antibacterial agent. The percentage of biofilm formation (BF) was determined by the formula: %BF = (Control OD570 nm / Test OD570 nm) × 100%. Culture without adding any honeys was used as a control and the wells containing the culture medium alone were used as blanks.

### 3.6. Statistical Analysis

All measurements were made in triplicate. For the data obtained, mean values and standard deviations were calculated. Significant differences were checked using a two-way analysis of variance followed by a *t*-test. The correlations between the obtained parameters were analyzed by a Pearson coefficient (r). All calculations were made using Statistica 13.3 software (StatSoft, Tulsa, OK, USA).

## 4. Conclusions

A significant dose-dependent increase in the content of bioactive ingredients and antioxidant capacity was found for each plant superfruits-enriched honey in comparison with the raw control honey. The highest increase was obtained for the addition of sea buckthorn leaves, followed by black raspberry, black currant, wild rose, and barberry fruits.

Honey with the addition of sea buckthorn leaves inhibited the growth of planktonic *P. aeruginosa, S. aureus* and *K. pneumoniae* bacteria. The weaker activity for honeys with black raspberry and blackcurrant fruits was observed against the last two strains only. Furthermore, honey flavored with sea buckthorn leaves and black raspberry fruits had a dose-dependent effect on the inhibition of biofilm formation by *S. aureus* ATCC 6538 in sub-MICs concentrations.

It can be concluded that enriching honey with plant superfoods significantly improves the antioxidant properties of rapeseed honey in each case; therefore, such functional products can be recommended as a source of exogenous antioxidants supporting the internal antioxidant defense system.

## Figures and Tables

**Table 1:** Phenolic compounds content and antioxidant capacity of plant-flavored honeys (fresh sample).

Honey Sample	TPC [mg GAE/100 g]	TFC [mg QE/100 g]	FRAP [μmol TE/100 g]	DPPH [μmol TE/100 g]	ABTS [μmol TE/100 g]
Control rapeseed honey	35.56 ± 0.21	9.16 ± 0.43	59.36 ± 0.31	52.66 ± 5.82	298.63 ± 2.15
Black raspberry (2%)	78.27 ± 2.10 *	15.27 ± 0.00	301.61 ± 12.32 *	191.03 ± 5.81 *	825.23 ± 26.87 *
Black raspberry (4%)	150.45 ± 3.58 *	52.67 ± 0.65 *	679.68 ± 2.28 *	412.81 ± 2.12 *	1091.94 ± 0.00 *
Blackcurrant (2%)	82.44 ± 0.84 *	24.28 ± 3.67	280.97 ± 15.05 *	156.25 ± 1.06 *	646.66 ± 51.58 *
Blackcurrant (4%)	159.82 ± 0.43 *	30.69 ± 0.65 *	589.36 ± 15.97 *	362.69 ± 6.48 *	1101.82 ± 1.08 *
Sea buckthorn—fruit (2%)	46.87 ± 0.21	34.50 ± 0.86 *	102.58 ± 17.34 *	111.00 ± 2.64 *	373.86 ± 56.96
Sea buckthorn—fruit (4%)	105.21 ± 3.16 *	99.77 ± 2.59 *	370.00 ± 14.14 *	168.22 ± 5.29 *	792.55 ± 19.34 *
Sea buckthorn—fruit powder (2%)	56.40 ± 0.63	10.69 ± 1.73	131.94 ± 7.76 *	97.16 ± 3.17 *	417.93 ± 22.57 *
Sea buckthorn—fruit powder (4%)	82.89 ± 3.31 *	54.81 ± 1.08 *	275.48 ± 10.95 *	161.86 ± 7.93 *	750.24 ± 12.90 *
Sea buckthorn—leaf (2%)	192.41 ± 4.00 *	29.62 ± 0.43 *	1043.22 ± 33.76 *	562.03 ± 47.07 *	1057.75 ± 37.61 *
Sea buckthorn—leaf (4%)	616.82 ± 5.26 *	76.64 ± 2.16 *	3543.23 ± 62.04 *	1852.72 ± 13.22 *	2200.60 ± 2.15 *
Elderberry—flower (2%)	92.41 ± 0.63 *	51.45 ± 3.26 *	273.87 ± 8.67 *	191.78 ± 2.64 *	775.07 ± 9.67 *
Elderberry—flower (4%)	211.31 ± 1.26 *	82.75 ± 2.16 *	638.71 ± 5.47 *	470.02 ± 4.76 *	1711.24 ± 6.45 *
Apple powder (2%)	40.77 ± 2.53	11.76 ± 0.22	81.29 ± 1.82 *	61.63 ± 1.59	349.54 ± 5.37
Apple powder (4%)	59.08 ± 7.78	17.41 ± 0.00	133.87 ± 19.61 *	112.12 ± 2.12 *	501.52 ± 18.27 *
Rose hip (2%)	165.00 ± 7.01 *	23.52 ± 2.06 *	851.04 ± 4.80 *	288.40 ± 6.36 *	1374.43 ± 44.41 *
Rose hip (4%)	306.11 ± 6.96 *	43.64 ± 4.59 *	1242.12 ± 45.00 *	360.46 ± 7.54 *	2051.75 ± 82.23 *
Barberry (2%)	79.08 ± 1.34	16.63 ± 1.89	309.01 ± 15.20 *	141.95 ± 4.24 *	404.71 ± 12.67 *
Barberry (4%)	143.20 ± 1.52 *	25.32 ± 3.06	600.13 ± 10.01 *	265.90 ± 23.26 *	523.74 ± 18.84 *

*—statistically significant difference compared to control rapeseed honey (*p* < 0.05).

**Table 2:** Minimal inhibitory concentration (MIC) values determined for the prepared honeys.

Tested Sample	MIC [mg/mL] *			
	*Escherichia coli* ATCC 10536	*Pseudomonas aeruginosa* ATCC 15442	*Staphylococcus aureus* ATCC 6538	*Klebsiella pneumoniae* ATCC 13883
Control rapeseed honey	NA	NA	NA	NA
Black raspberry (4%)	NA	NA	250 (17.85% *v/v*)	250 (17.85% *v/v*)
Blackcurrant (4%)	NA	NA	250 (17.85% *v/v*)	250 (17.85% *v/v*)
Sea buckthorn—fruit (4%)	NA	NA	NA	NA
Sea buckthorn—fruit powder (4%)	NA	NA	NA	NA
Sea buckthorn—leaf (4%)	NA	250 (17.85% *v/v*)	125 (8.93% *v/v*)	125 (8.93% *v/v*)
Elderberry—flower (4%)	NA	NA	NA	NA
Apple powder (4%)	NA	NA	NA	NA
Rose hip (4%)	NA	NA	NA	250
Barberry (4%)	NA	NA	NA	NA
Chloramphenicol	3.9 × 10^−3^	0.25	7.8 × 10^−3^	7.8 × 10^−3^
Gentamicin	1.9 × 10^−3^	1.9	0.97 × 10^−3^	0.2 × 10^−3^

NA—no antibacterial activity; *—values in parentheses have been converted to % *v/v*, taking into account the density of the honey.

## Data Availability

The data presented in this study are available in the article.
